# Combining CRP testing and patient information leaflets to safely reduce antibiotic use for acute respiratory tract infections in adults: Protocol for the 2CARE randomised controlled trial in Kyrgyz primary care

**DOI:** 10.1371/journal.pone.0345747

**Published:** 2026-04-10

**Authors:** Azamat Akylbekov, Elvira Isaeva, Maamed Mademilov, Volkert Siersma, Torsten Risør, Sif Helene Arnold, Jesper Kjærgaard, Nandini Sreenivasan, Ahmad Wesal Zaman, Talant Sooronbaev, Rune Munck Aabenhus

**Affiliations:** 1 Department of Pulmonology, National Centre of Cardiology and Internal Medicine named after academician M. Mirrakhimov, Bishkek, Kyrgyzstan; 2 Department of Allergology, National Centre of Maternity and Childhood Care, Bishkek, Kyrgyzstan; 3 The Research Unit for General Practice and Section of General Practice, Department of Public Health, University of Copenhagen, Copenhagen, Denmark; 4 Unit for children and adolescents with infections or organ-disease, Copenhagen University Hospital, Rigshospitalet, Copenhagen, Denmark; 5 International Centre for Antimicrobial Resistance Solutions (ICARS), Copenhagen, Denmark; University of California at Davis, UNITED STATES OF AMERICA

## Abstract

**Background:**

Antimicrobial resistance (AMR) is a major health challenge in Kyrgyzstan, where acute respiratory tract infections (ARTIs) are common and frequently treated with antibiotics. Although C-reactive protein (CRP) point-of-care testing can support rational prescribing, there is no consensus on safe CRP thresholds for adults. Patient information leaflets (PILs) may further reduce inappropriate antibiotic use by improving understanding of ARTIs and addressing expectations for antibiotics. The 2CARE trial evaluates whether combining PILs with CRP testing can safely reduce antibiotic use among adults with ARTIs in Kyrgyz primary care.

**Methods:**

2CARE is a multicentre, open-label, individually randomised controlled trial conducted in 15 primary healthcare centres in Kyrgyzstan. Adults aged 18–70 years with acute respiratory symptoms (<14 days) are randomised 1:1 to receive a PIL or no PIL, and independently 1:1:1 to one of three CRP cut-off levels (20, 40, or 60 mg/L), yielding six parallel groups. Primary outcomes are: (1) total antibiotic use within 21 days, and (2) hospital admission within 21 days. Secondary outcomes include baseline antibiotic prescribing, re-consultations, recovery, hospital referral, antiviral use, and mortality. Analyses will follow the intention-to-treat principle, with per-protocol analysis for non-inferiority, using regression models and generalized estimating equations. A total sample of 1,050 participants provides 90% power for both primary comparisons.

**Discussion:**

This trial will generate evidence on the effectiveness of PILs in reducing antibiotic use and establish safe CRP thresholds for adults with ARTIs in a low-resource setting. Findings will inform antimicrobial stewardship policies and guide national CRP implementation in Kyrgyzstan.

**Trial registration:**

ClinicalTrials.gov Identifier: NCT07261969.

## Introduction

Antimicrobial resistance (AMR) is an escalating global public health threat that undermines the effectiveness of antibacterial therapies and contributes to rising morbidity, mortality, and healthcare costs [1–3]. The burden is highest in low- and middle-income countries (LMICs), where infectious diseases are more prevalent, antibiotic access is less regulated, and stewardship infrastructure remains limited [4]. Reducing unnecessary antibiotic use is therefore a key global priority for mitigating AMR and preserving the efficacy of existing treatments.

Acute respiratory tract infections (ARTIs) are among the most common reasons for healthcare attendance and antibiotic prescribing worldwide [5–7]. Most ARTIs are viral and self-limiting, yet diagnostic uncertainty and entrenched expectations among patients and clinicians often lead to inappropriate empirical antibiotic treatment [8,9]. Scalable interventions that address both the diagnostic and behavioural drivers of antibiotic use are urgently needed, particularly in LMIC primary care systems. Patient demand, limited consultation time, cultural norms, and clinicians’ perceptions about expectations frequently contribute to unnecessary prescribing [10].

C-reactive protein (CRP) point-of-care testing has been widely studied as a tool to support rational antibiotic prescribing for ARTIs. CRP testing can reduce unnecessary antibiotic use by helping clinicians distinguish between likely viral and bacterial infections and by providing objective evidence to support non-prescription decisions [11–13]. However, there is no consensus on which CRP cut-off values safely guide antibiotic decisions in adults. Existing studies vary substantially in their recommended thresholds, and most evidence originates from high-income countries [14]. These thresholds may not be directly applicable to LMIC settings, where patient characteristics, access to care, pathogen profiles, and risks associated with delayed treatment differ.

This challenge is particularly relevant in Kyrgyzstan. Antibiotics can be purchased over the counter without prescription, and self-medication is widespread [15,16]. These patterns shape patient expectations and influence clinicians’ prescribing decisions, even when bacterial infection is unlikely. Additional contextual pressures on clinicians, including workload [17], time constraints, diagnostic uncertainty, and sociocultural expectations, further contribute to inappropriate antibiotic prescribing [18]. In many rural areas, healthcare workers (HCWs) manage ARTIs empirically due to limited diagnostic capacity, frequent staff shortages, and geographical isolation. Such conditions make point-of-care diagnostics like CRP testing highly valuable for supporting clinical decision-making [11]. At the same time, limited health literacy and misconceptions about antibiotics may reduce the behavioural impact of diagnostic guidance alone [19]. Patients may still seek antibiotics independently, even when clinicians advise against their use.

Educational tools such as patient information leaflets (PILs), available in Kyrgyz and Russian, offer an accessible way to counter these behavioural drivers [20]. PILs explain the natural course of ARTIs, outline when antibiotics are and are not required and provide safety-netting advice [21]. Evidence from primary-care trials shows that written information can reduce antibiotic demand, improve patient satisfaction, and reduce re-consultation without compromising safety [22,23]. In settings with unrestricted antibiotic access, PILs may therefore enhance the effectiveness of diagnostic interventions like CRP testing by aligning patient expectations with clinically appropriate management.

Our team has previously demonstrated the feasibility and impact of CRP testing in Kyrgyzstan through the COORDINATE randomised controlled trial in paediatric primary care. CRP testing reduced antibiotic prescribing by 24 percentage points (36% vs 60%) without increasing hospitalisations or delaying recovery [24,25]. These findings have catalysed national interest in scaling CRP testing more widely. However, evidence on CRP thresholds for adults in Kyrgyzstan is lacking, and no studies have evaluated the combined effect of diagnostic support and structured patient education in this context.

The 2CARE trial addresses this gap by evaluating whether PILs reduce total antibiotic use and by testing three CRP thresholds (20, 40, and 60 mg/L) to identify safe and effective cut-offs for guiding antibiotic prescribing in adults with ARTIs in Kyrgyz primary care.

## Methods

### Patient and public involvement

No patients or members of the public were involved in setting the research question, defining the outcome measures, designing the study, or planning the recruitment, conduct, or dissemination of this trial.

### Study design

**Fig 1:**
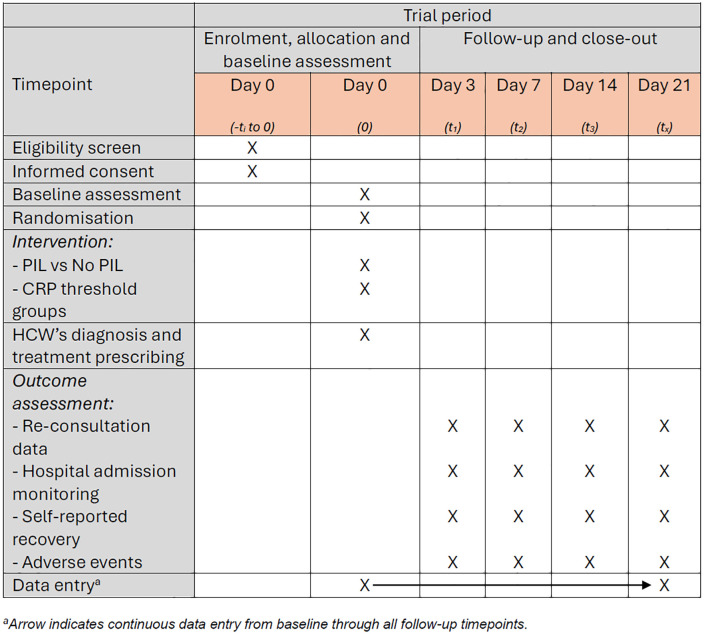
SPIRIT schedule of enrolment, interventions and follow-up assessments for the 2CARE trial.

The 2CARE study will be a multicentre, open-label, individually randomised controlled clinical trial with 21-day blinded follow-up. No unblinding procedures are required because follow-up assessors collect self-reported data only and have no access to allocation information. The overall schedule of enrolment, interventions and assessments follows the SPIRIT recommendations [26] and is presented in Fig 1.

The trial will evaluate two complementary interventions designed to optimise antibiotic use among adults presenting with ARTIs in Kyrgyz primary care:

(1) the use of PILs that explain what the condition is, provide safety-netting, and indicate whether antibiotics are needed, and
(2) the application of different CRP thresholds to guide safe antibiotic prescribing decisions.

**Fig 2:**
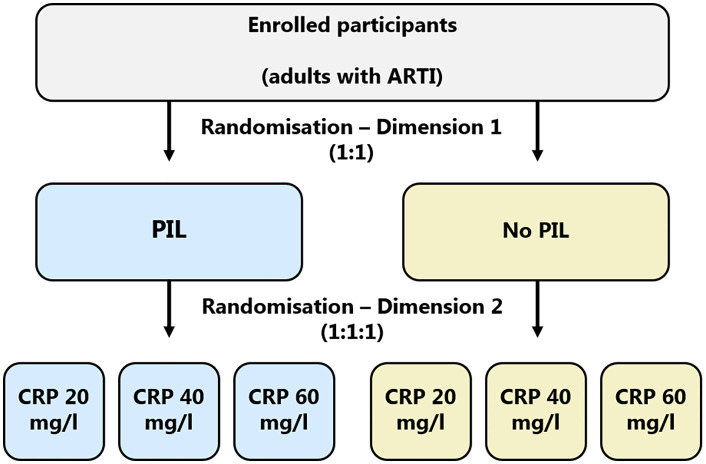
The randomisation scheme. Participants are randomised simultaneously along two independent dimensions: (1) to receive a patient information leaflet (PIL) or no PIL (1:1), and (2) to one of three CRP thresholds (20, 40 or 60 mg/L; 1:1:1).

Randomisation will be performed using site-stratified block randomisation, with separate randomisation lists generated for each participating primary healthcare centre to ensure balance across sites. Participants will be randomised in a 1:1 ratio to receive either a PIL or no PIL, and, in parallel, in a 1:1:1 ratio to one of three CRP cut-off levels (20, 40, or 60 mg/L). This factorial design will generate six parallel groups, allowing independent assessment of both interventions and their potential interaction. Follow-up outcome assessments will be conducted by research staff who are blinded to treatment allocation, with calls at Days 3, 7, 14 and 21 assigned through an internal rotation schedule to minimise observer bias. The factorial allocation scheme is shown in Fig 2.

The trial is designed in accordance with the SPIRIT recommendations for study protocols [26] and will be reported following the CONSORT guidelines for randomised clinical trials [27].

### Study setting

The trial will be conducted in 15 primary healthcare centres (PHCs) across Kyrgyzstan, including one urban site in Bishkek and 14 rural sites in the Chui and Naryn regions. These centres reflect the diversity of the Kyrgyz primary care system, which operates as the first point of contact for most patients and includes both densely populated urban areas and remote mountain villages. The participating PHCs range from small rural health posts staffed only by nurses to larger family medicine centres with multiple physicians and nursing staff [24,25].

Most facilities have only basic diagnostic tools and limited laboratory capacity. Simple tests such as urinalysis or complete blood counts are often unavailable, and diagnostic decisions are frequently based solely on clinical assessment. HCWs involved in the trial will include family doctors and nurses, reflecting current staffing patterns in Kyrgyz primary care.

### Participants

Eligible participants will be adults aged 18–70 years presenting to participating PHCs with symptoms of ARTI during two consecutive winter–spring periods (2025–2026). Screening will be conducted on weekdays by trained research assistants (RAs) before the routine consultation, either in waiting areas or inside consultation rooms. RAs will perform eligibility screening, randomisation, and baseline data entry, while HCWs will conduct CRP testing and deliver the PIL intervention. Baseline data will include age, sex, smoking status and relevant chronic conditions (e.g., diabetes, chronic heart disease, chronic lung disease) to allow adjustment for potential confounding in the analyses.

Participants will be eligible for inclusion if they have at least one acute respiratory symptom, such as cough, shortness of breath, sore throat, nasal congestion, or wheezing, with or without fever lasting less than two weeks, and are able and willing to provide written informed consent and comply with study procedures.

Exclusion criteria include severe illness requiring urgent referral or hospitalisation, terminal illness, known immunosuppression or severe chronic disease (e.g., HIV, hepatic or renal failure, neoplastic disease, long-term systemic steroid use), inability to participate in follow-up (e.g., lack of telephone), antibiotic use within 24 hours before enrolment, pregnancy, or refusal to consent. No additional eligibility criteria are applied at the site level beyond the requirement that participating PHCs employ HCWs trained in CRP POCT and PIL delivery.

All eligible participants will receive verbal and written information about the study before enrolment and written informed consent will be obtained. The research team will not interfere with emergency referrals, unplanned examinations, or hospital admissions if participants require urgent medical attention.

### Interventions

All study procedures related to the interventions will be delivered by trained healthcare workers (HCWs) at the participating primary healthcare centres.

During the baseline consultation, the HCW will perform a CRP POCT using the QuickRead Go device (Aidian, Espoo, Finland) [28]. Capillary blood will be obtained via finger prick, and the test will be conducted following the manufacturer’s standardised operating procedures. The CRP value will be available within approximately two minutes, recorded in the case report form (CRF), and communicated to the participant in clear, non-technical language. The HCW will apply the CRP threshold assigned through randomisation (20, 40 or 60 mg/L) to guide the antibiotic prescribing decision. All prescribing decisions remain the responsibility of the HCW.

CRP point-of-care testing guidance in adults with ARTIs presenting in primary care distinguishes a low-risk zone below 20 mg/L, where antibiotics are not routinely recommended, and an intermediate range in which prescribing decisions depend on clinical assessment [29]. We therefore selected 20 mg/L as a conservative rule-out threshold and 40 mg/L and 60 mg/L as two pragmatic values within the intermediate range to explore the balance between reduced antibiotic use and safety [30]. As CRP-guided prescribing thresholds have not previously been evaluated in adult primary care in Kyrgyzstan, higher thresholds were not included.

Participants allocated to the PIL arm will receive the leaflet directly from the HCW at the end of the consultation. Two complementary PILs developed by the World Health Organisation (WHO) will be used together as a single intervention in this trial. One leaflet (“What you need to know about antibiotics”) provides an explanation of when antibiotics are necessary or unnecessary, emphasises that most ARTIs are viral, outlines common side effects of antibiotics including the risk of antimicrobial resistance, and encourages discussion with the healthcare worker. The second leaflet is consultation-linked (a “prescription for non-prescribing antibiotics”) and includes structured space for documenting the clinical decision, symptom-based self-care advice, and safety-netting instructions describing warning signs that require urgent medical attention. Both leaflets were translated and culturally adapted into Kyrgyz and Russian in collaboration with the WHO, use plain language and simple visual design, and are delivered using a standardised script.

Before participant enrolment begins, all HCWs will complete structured training sessions covering the technical performance of CRP POCT, interpretation of CRP results, and application of the three randomised CRP thresholds for initiating or withholding antibiotic therapy. During training, healthcare workers will be instructed to use CRP results primarily in a binary manner relative to the assigned cut-off (below vs above threshold) when considering antibiotic prescribing. The procedures of performing the CRP test and accompanying results may be shared with participants. The absolute CRP value will not be used to modify the assigned threshold, although clinical judgement may still prompt referral or additional evaluation if severe illness is suspected. Training will also include communication skills and standardised delivery of the PIL intervention. Sessions will be conducted in Kyrgyz or Russian, depending on site preference. Refresher training will be provided when necessary to maintain protocol adherence throughout the study period.

CRP testing will be performed using the QuickRead Go point-of-care device, with HCWs trained to follow the manufacturer’s instructions consistently. Fidelity will be supported through ongoing monitoring of CRFs, periodic reinforcement of key procedures and continuous communication between the study team and participating sites.

All participants will be followed for 21 days after the baseline consultation. Follow-up assessments will be conducted via structured telephone interviews on Days 3, 7, 14 and 21. To minimise observer bias, all follow-up calls will be performed by research staff who are blinded to treatment allocation and not involved in baseline assessment or intervention delivery. Calls will be distributed according to an internal rotation schedule to ensure that, where feasible, different blinded assessors contact each participant at different timepoints. Follow-up interviews will collect data on symptom progression, antibiotic consumption, healthcare utilisation, re-consultations, hospital admissions and adverse events. Allocated interventions are not modified after randomisation. Participants whose clinical condition worsens receive routine medical care, including referral or hospitalisation, according to national guidelines. All other aspects of routine clinical care, including symptomatic treatment, are permitted.

### Safety considerations

CRP POCT testing is widely used in primary care and is considered safe, requiring only a capillary finger-prick sample. PILs are non-invasive educational materials and pose no direct risk. All participants will receive usual clinical care, and healthcare workers may refer patients for additional evaluation or hospitalisation at any time based on clinical judgement, irrespective of trial allocation. Serious adverse events will be reported to the safety board within 24 hours and reviewed according to predefined procedures. The safety board may recommend temporary suspension or early termination of the trial if unacceptable risks are identified.

### Outcomes

The first primary outcome will be total antibiotic use within 21 days, defined as any antibiotic consumption during the follow-up period, whether prescribed by a HCW or obtained independently through over-the-counter purchase or similar sources. This outcome primarily reflects the effect of the PIL on participants’ and clinicians’ antibiotic-related behaviour.

The second primary outcome will be hospital admission within 21 days, serving as the main safety indicator. It reflects the potential risks associated with higher CRP thresholds and the possibility of delayed or missed treatment.

Secondary outcomes include antibiotic prescribing at the baseline consultation and between CRP groups, self-reported recovery, antibiotic use initiated outside of healthcare visits, antiviral treatment, re-consultation, hospital referral at the baseline visit, and mortality.

All outcomes will be assessed over a 21-day follow-up period. Follow-up interviews will be conducted by telephone on days 3, 7, 14, and 21 by trained RAs who will remain blinded to participants’ allocation.

### Sample size and power calculation

For the primary effectiveness outcome of total antibiotic use, it is expected that 40% of participants in the control group (no PIL) will use antibiotics within 21 days. A 10% reduction in the intervention group is considered clinically relevant. Assuming a two-sided significance level of 5% and 90% power to detect this difference, 506 participants will be required per arm, giving a total of 1,012 participants for this comparison.

For the primary safety outcome (hospital admission within 21 days), it is anticipated that about 5% of participants will require hospitalisation in the reference group. A difference larger than five percentage points will define the non-inferiority margin. To conclude non-inferiority with 90% power and a two-sided 5% significance level, 326 participants will be required in each CRP threshold group, giving a total of 978 participants.

The study is not powered to formally test interaction effects between the PIL intervention and CRP threshold allocation. Any interaction analyses will therefore be considered exploratory.

To cover both primary outcomes and allow for an estimated 5% loss to follow-up, the final planned sample size will be 1,050 participants. One interim analysis will be performed when 500 participants have completed follow-up. The planned interim analysis is intended for safety monitoring only and not for formal hypothesis testing; therefore, no adjustment to the overall sample size or significance level was applied.

### Study timeline

Recruitment began on 19 December 2024. At the time of manuscript submission, recruitment was ongoing and approximately 50% of the target sample size had been enrolled. Recruitment is expected to be completed by May 2026, with data collection concluding after completion of follow-up for the final participant. Data cleaning, database lock and primary analyses are expected to be completed by late 2026. Dissemination of trial results is planned for 2027–2028.

### Statistical analysis

All primary analyses will follow the intention-to-treat (ITT) principle, including all randomised participants as allocated. For the safety endpoint (hospital admission within 21 days), a per-protocol (PP) analysis will also be conducted. Non-inferiority will be concluded only if the prespecified non-inferiority margin is not exceeded in both the ITT and per-protocol analyses. Given that the interventions are prescribing and communication strategies rather than treatment exposures, and that the relevant exposure is adherence to the assigned decision rule, a conventional ‘as-treated’ safety population is not considered applicable.

The effectiveness outcome (total antibiotic use within 21 days) will compare PIL versus no-PIL groups using risk differences and risk ratios with 95% confidence intervals. The safety outcome (hospital admission within 21 days) will compare the three CRP threshold groups (20, 40, 60 mg/L) using a non-inferiority margin prespecified in the Statistical Analysis Plan. Baseline characteristics will be summarised using appropriate descriptive statistics, and between-group balance will be assessed to illustrate the performance of randomisation.

For the primary analyses, regression models will be fitted to estimate absolute and relative risks while adjusting for key baseline characteristics, including age, sex and the presence of relevant chronic conditions (e.g., diabetes, chronic heart disease, chronic lung disease), to account for potential differences in background inflammatory status. For repeated follow-up measures (e.g., antibiotic use and symptoms across Days 3–21), generalised estimating equations (GEE) with robust standard errors will be used to account for within-participant correlation over time. Logistic regression models will be used for binary outcomes measured at a single timepoint. All adjusted models will include the orthogonal randomisation factor to appropriately reflect the factorial design.

As a sensitivity analysis, we will repeat the primary comparisons after excluding participants with selected chronic conditions known to be associated with low-grade CRP elevation to assess the robustness of the findings. Additional exploratory analyses will estimate potential interaction effects between PIL allocation and CRP threshold, acknowledging that the study is not powered for interaction testing.

Missing outcome data will be handled using complete-case analyses for the primary comparisons, supplemented by multiple imputation under missing-at-random assumptions in sensitivity analyses. All statistical tests will be two-sided except for the non-inferiority comparison, which will use a one-sided significance level consistent with the prespecified margin.

All analyses will be conducted using R [31] and SAS [32], following the prespecified and publicly archived Statistical Analysis Plan developed in collaboration with the study biostatistician. Missing outcome data are handled using complete-case analysis, with additional sensitivity analyses using multiple imputation if appropriate.

### Safety and oversight

A safety board consisting of the principal investigator, a biostatistician, and two independent academic members (one Kyrgyz and one Danish) will review all serious adverse events (SAEs) and be presented with the interim safety report. The board may recommend temporary suspension or termination of the trial in case of safety concerns.

### Data collection and management

All study data will be collected using structured case report forms (CRFs) developed specifically for this trial. Participant information and baseline data will be recorded on paper CRFs by trained RAs, who will conduct eligibility screening, provide study information, obtain written informed consent and perform randomisation. HCWs will complete only the clinical components of the CRF related to the consultation (e.g., CRP result, clinical assessment, and antibiotic prescribing decision). PILs will be managed and distributed by HCWs as part of the intervention delivery. Each CRF will include a unique participant identification number to ensure traceability without the use of personal identifiers.

Completed CRFs will be checked on site by the RAs for completeness and internal consistency before being transferred to the coordinating office in Bishkek. Subsequently, data will be double-entered into a secure electronic database by two independent data clerks to minimise entry errors. Discrepancies between entries will be identified and resolved through source verification by the data manager.

Data quality will be monitored throughout the study through regular remote and on-site checks by the coordinating research team. Any missing or implausible data points will be queried and resolved before database lock. All paper documents will be stored in locked cabinets accessible only to authorised personnel, and electronic data will be stored on password-protected computers with restricted access.

Major protocol deviations, such as failure to apply the assigned intervention, incorrect CRP threshold use, or incomplete follow-up affecting the primary outcome – will be documented and reviewed.

At the end of the trial, the final cleaned and anonymised dataset will be archived securely and may be made available to other researchers upon reasonable request and approval by the principal investigator and the Ethics Committee.

## Ethics and dissemination

### Ethical approval

The study protocol was approved by the Ethics Committee of the National Centre of Cardiology and Internal Medicine named after academician M. Mirrakhimov (Ref. No 10, 27 November 2024).

### Ethics and data protection

Written informed consent will be obtained from all participants prior to enrolment. Participant confidentiality will be strictly protected and maintained. No ancillary biological samples are collected in this trial. All data will be handled in accordance with Kyrgyz National data-protection regulations and where relevant also the EU General Data Protection Regulation (GDPR).

### Dissemination plan

Findings will be disseminated through peer-reviewed open-access publications, conference presentations, and national stakeholder workshops. Results will be shared with participating sites and the Ministry of Health of Kyrgyzstan. Summaries of results will be provided in accessible language to participating PHCs and, where feasible, to study participants.

## Discussion

Randomised controlled trials remain uncommon in Kyrgyzstan, where limited research capacity and logistical constraints often hinder the implementation of complex study designs. Building upon experience from the COORDINATE paediatric trial on CRP POCT for ARTIs in children [27], the 2CARE trial represents a major step towards expanding evidence-based antimicrobial stewardship interventions to the adult population within the INSTALL study.

Although much of the evidence on antibiotic stewardship has been generated in high-income countries, where healthcare infrastructures, patient expectations and patterns of antibiotic access differ substantially, several recent reports from Central Asia, including a nationwide evaluation of antibiotic consumption in Kazakhstan [33] and a WHO situational overview of antibiotic use in Central Asian, Caucasus and Eastern European countries [34,35], highlight similar regional challenges. However, rigorous randomised trials from this part of the world remain scarce, particularly in primary care settings. Against this backdrop, the 2CARE trial provides an important opportunity to generate locally relevant evidence on diagnostic and behavioural stewardship interventions in Kyrgyzstan, where widespread over-the-counter antibiotic availability, self-medication habits and limited diagnostic capacity underscore the need for locally validated CRP thresholds and culturally adapted communication tools.

The 2CARE trial combines diagnostic guidance through CRP POCT with patient education via PILs, addressing both clinical and behavioural determinants of antibiotic misuse. In Kyrgyzstan, where antibiotics are commonly perceived as the default response to respiratory symptoms, PILs may serve as a low-cost and scalable way to shift expectations, improve understanding and support adherence to professional advice when CRP levels indicate a likely viral infection. The combined effect of point-of-care diagnostics and structured communication has not previously been evaluated in a Central Asian context, making 2CARE one of the first pragmatic trials to explore both components simultaneously. This dual approach may generate locally relevant evidence on safe CRP thresholds for adults and on how patient understanding influences antibiotic demand and treatment adherence.

Although the trial is not powered to formally assess interaction effects between the PIL intervention and CRP threshold allocation, exploratory analyses may generate hypotheses about how communication and diagnostic guidance interact in antibiotic decision-making. Future studies could be designed to explicitly evaluate patient information materials that explain the role and limitations of CRP testing and to assess whether such integrated communication further enhances shared understanding and appropriate antibiotic use in primary care.

Several limitations merit consideration. The trial is open-label because blinding of health workers and participants is not feasible for behavioural and diagnostic interventions of this type. Although outcome assessors remain blinded, awareness of allocation could still influence clinical behaviour or self-reporting. Variation across primary healthcare centres may also introduce heterogeneity. While RAs perform screening, consent procedures and follow-up, the clinical interventions (CRP testing, interpretation and provision of the PIL) are delivered by HCWs, whose experience, workload and local workflows differ across settings. In smaller rural health posts staffed mainly by nurses and in larger urban family medicine centres, the degree of support needed to maintain protocol adherence may vary. Minor losses to follow-up or under-reporting of over-the-counter antibiotic use may also occur. Furthermore, some chronic conditions are associated with low-grade elevations in CRP, which could influence threshold-based prescribing decisions; this will be explored through adjusted and sensitivity analyses. These challenges are intrinsic to pragmatic field studies in resource-limited primary care settings but will be mitigated through rigorous training, supervision and predefined analytical strategies.

Kyrgyz primary care operates within a system where mechanisms for quality development, continuing professional education and adoption of new diagnostic tools are still evolving [36]. This makes the integration of CRP POCT and structured communication materials particularly relevant in this setting. Yet these challenges are not unique to Kyrgyzstan. Many low- and middle-income countries face similar constraints, including limited research infrastructure, variable health worker training, restricted access to diagnostics and widespread over-the-counter antibiotic use. By generating contextually grounded evidence on both diagnostic and behavioural stewardship strategies, the 2CARE trial has the potential to inform not only national implementation pathways in Kyrgyzstan but also broader efforts to advance safe and rational antibiotic use in comparable primary care systems across LMICs.

## Supporting information

S1 File SPIRIT checklist. (PDF)

S2 File Patient information leaflet 1. (PDF)

S3 File Patient information leaflet 2. (PDF)

S4 File Study protocol. (PDF)
