# Supplementary material for: Combining CRP testing and patient information leaflets to safely reduce antibiotic use for acute respiratory tract infections in adults: Protocol for the 2CARE randomised controlled trial in Kyrgyz primary care
Source: PLoS One. 2026 Apr 10;21(4):e0345747. doi: 10.1371/journal.pone.0345747 (PMC13068273; doi:10.1371/journal.pone.0345747)
Supplement: S3 File — (PDF) [file pone.0345747.s003.pdf]

What you need to know about antibiotics

- **Antibiotics are only effective to treat certain bacterial infections.**
  Many infections, also the ones caused by bacteria, may clear up on their own without antibiotics. Your doctor will ask for certain symptoms and judge whether antibiotics will be needed. Antibiotics do not work for viral infections (e.g. a bad cold, the flu or Covid-19).
- **In many cases, your body's immune system is strong enough.**
  A healthy body generally fights simple infections with great efficiency. Many of the symptoms we experience when being sick are signs of an active immune system fighting the infection. That could be fever, for example.
- **Incorrect intake can cause resistance to antibiotics.**
  Antibiotics need to be taken in correct dosages and intervals. If you interrupt the treatment or take antibiotics irregularly, some of the bacteria might survive and develop resistance to the antibiotic.
- **Antibiotics can sometimes produce harmful side effects.**
  Some persons experience unwanted effects such as diarrhea or stomach ache when taking antibiotics, others might develop unpleasant allergic reactions such as an itchy skin rash. This should not stop you from taking antibiotics when needed, but why deal with this, when you have an infection that doesn't demand antibiotics?

What is AMR?

AMR stands for antimicrobial resistance. Antimicrobial resistance is a defense mechanism developed by bacteria and other microorganisms to protect themselves from being killed by medication. AMR develops over time, but sometimes even over a short treatment course.

AMR can happen in all bacteria, the pathogenic ones and the good ones. You might not even notice that you are carrying resistant bacteria with you, but suddenly and unexpectedly you can fall sick. Or you can pass them on to persons who are more vulnerable. This can be someone you love. Therefore you should be careful in the way you use antibiotics.

Please speak to your doctor for further information.

HANDLE ANTIMICROBIALS WITH CARE

© World Health Organization 2022. Some rights reserved.
This work is available under the CC BY-NC-SA 3.0 IGO license

Patient Information Leaflet

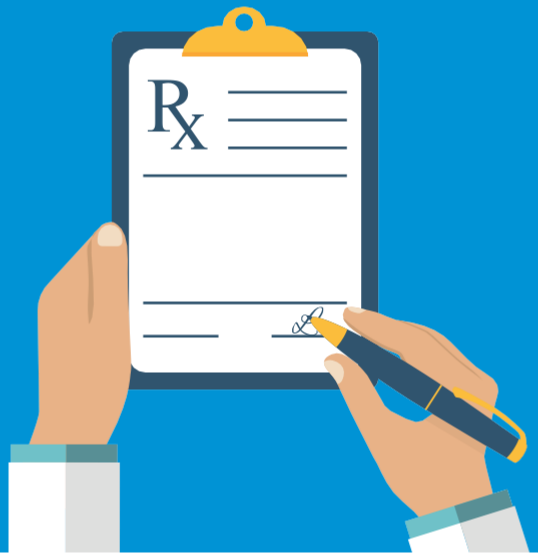

Patient

Name	
Age	
Date	

For GPs to fill out with their patients at consultation

World Health Organization
REGIONAL OFFICE FOR Europe

Respiratory infections

Main symptoms today:

- ☐ Sore throat
- ☐ Angina
- ☐ Ear pain
- ☐ Trouble hearing
- ☐ Pressure over sinuses
- ☐ Running nose and watery eyes
- ☐ Morning cough
- ☐ Cough with expectoration
- ☐ Muscular and joint pains
- ☐ Stiff neck
- ☐ Slight fever (below 38.5°C)
- ☐ Other

____

____

Diagnosis given at this consultation:

Recommendations:

- ☐ Yes, antibiotic given.
- ☐ No, antibiotic not given.

Dosage and duration: ____

____

____

____

What can you do?

- For a sore throat: eat ice cream, ice cubes or throat lozenges.
- For a stuffy nose: saline nasal spray or drops.
- For fever and pain relief: paracetamol or ibuprofen.
- Get as much rest as possible and drink plenty of fluids.
- Take a hot or cold shower if it relieves you and take off the blankets, when you feel very hot.
- Wash your hands frequently and try to stay home as not to infect others.

What to watch for/ when you should seek medical help again:

- Fever with chills that does not respond to antipyretics as suggested in the green box.
- Fever that comes and goes for several days without improvement.
- Chest pain and difficulties breathing.
- If your family or friends find that you become confused or have slurred speech.
- If you have difficulty swallowing or are drooling.
- If you cough up blood.

Who should you call, when you get worse:

- Get a new appointment ____
- Go to out of hours medical service ________________________________
- Go to your local emergency departments ___________________________
