# Supplementary material for: Combining CRP testing and patient information leaflets to safely reduce antibiotic use for acute respiratory tract infections in adults: Protocol for the 2CARE randomised controlled trial in Kyrgyz primary care
Source: PLoS One. 2026 Apr 10;21(4):e0345747. doi: 10.1371/journal.pone.0345747 (PMC13068273; doi:10.1371/journal.pone.0345747)
Supplement: S4 File — (PDF) [file pone.0345747.s004.pdf]

CRP and patient information leaflets TO optimise AntibiotiC treatments for Adults with Respiratory tract infections in primary carE in Kyrgyzstan - part of the INSTALL Project

STUDY PROTOCOL

Version of the document 2.2 (10.12.2024)

Bishkek - Copenhagen 2024

PROJECT TITLE

CRP and patient information leaflets to optimise antibiotic treatments for adults with respiratory tract infections in primary care in Kyrgyzstan - <u>part of the INSTALL project</u>

BACKGROUND

Antibiotic resistance (AMR) is a significant public health issue both globally and in Kyrgyzstan, where the overuse and misuse of antibiotics have accelerated this crisis [1]. In Kyrgyzstan, antibiotics are often accessible over-the-counter without a prescription, contributing to widespread, uncontrolled usage and increasing resistance among common pathogens [2]. This rising resistance undermines the effectiveness of treatments, posing risks for both common infections and severe diseases, which leads to higher healthcare costs and mortality rates [3, 4].

In Kyrgyzstan, an interagency programme and action plan to tackle antibiotic resistance is under development, awaiting future approval. Evidence-based research is critical for informing this process. A 2011 study reviewed 251 medical records and 428 antibiotic prescriptions from 2007 and found inappropriate antibiotic therapy in 73.3% of the cases [5]. Despite attention from the Ministry of Health, resource shortages and a lack of research result in many issues remaining unaddressed. Kyrgyzstan is a member of the Central Asian and European Surveillance of Antimicrobial Resistance (CAESAR) network but does not yet report AMR data and faces shortcomings in training on rational antibiotic use among healthcare providers [6].

The Commonwealth of Independent States (CIS) is advancing a joint plan against AMR. Representatives from CIS countries met online in August 2021 to finalize a draft plan, which includes public education on AMR, increased vaccination, and training for healthcare and veterinary sectors. In 2022, a plan of joint actions of the CIS member states to combat AMR was adopted."

Globally, WHO warns of a potential "post-antibiotic era", with the overuse of antibiotics, especially for respiratory infections, posing significant risks [7]. In Kyrgyzstan, influenza and acute respiratory viral infections (ARVI) account for most infectious diseases, representing 66.6% of all reported cases on average and reaching up to 78% in certain years—exceeding the incidence of all other infections combined [8].

C-reactive protein testing - Improved diagnostics may work to reduce unnecessary antibiotic use. C-reactive protein (CRP) testing is a proven tool for improving antibiotic stewardship by helping clinicians determine when antibiotics are actually necessary, both in children [9] and adults [10]. By indicating the presence of likely bacterial versus viral infections, CRP testing assists the health care worker (HCW) in reducing unnecessary antibiotic prescriptions, making it an effective and safe approach to managing antibiotic use [11].

The COORDINATE trial [9] was launched in 2022 to assess the efficiency of C-reactive protein (CRP) a point of care test (POCT), as a diagnostic tool to aid health professionals in determine

if children with Respiratory Tract Infections (RTI) require antibiotics or not. This study in which children were individually randomised to have CRP POCT performed or not, was conducted in 14 primary health care facilities across the Chui (lowland) and Naryn (mountainous) areas of Kyrgyzstan. The CRP intervention in the study 1) effectively reduced antibiotic prescriptions, and 2) the reduced use of antibiotics did not negatively affect the safety of the children in terms of illness duration, recovery and risk of hospitalisation (paper in review). Distinguishing between viral and bacterial respiratory tract infections (RTIs) based solely on clinical examination poses significant challenges due to overlapping symptoms such as cough, fever, and malaise [12]. These common presentations make it difficult for clinicians to accurately identify the causative agent, often a viral or self-limiting bacterial infection, potentially leading to inappropriate treatment choices. CRP levels can serve as a valuable biomarker in this context, helping to assess the severity and prognosis of the infection [11]. Markedly elevated CRP levels often indicate a bacterial infection, suggesting that antibiotic treatment could alter the disease course and provide substantial benefits to the patient. Conversely, normal and mildly elevated CRP levels may point to a viral aetiology, where antibiotics would be ineffective and unnecessary. Therefore, CRP testing can aid in clinical decision-making, guiding more targeted and appropriate use of antibiotics, ultimately improving patient outcomes and reducing the risk of antibiotic resistance [13].

CRP POCT has been demonstrated to be an effective and safe marker for guiding antibiotic therapy in respiratory tract infections, as evidenced by systematic reviews, including those by Cochrane [11]. However, there remains a lack of consensus on the specific cut-off values for CRP levels in clinical practice. Although the COORDINATE study [9] established a threshold value of 10 mg/L for children, we still need working threshold values for the adult population in Kyrgyzstan.

Based on these results, the Ministry of Health of the Kyrgyz Republic decided to implement CRP POCT nationwide in all primary healthcare centres (HC), targeting all citizens, presenting with RTI. However, this the implementation plan requires additional studies to determine optimal and safe ways of using the CRP test in adults with RTIs and to successfully implement the programme.

Antibiotic-free leaflets, a type of Patient Information Leaflets (PILs) [14] are an educational tool aimed at reducing antibiotic misuse and thus combating antimicrobial resistance (AMR). These leaflets provide clear, accessible information for patients and caregivers on when antibiotics are necessary—and, importantly, when they are not. By explaining common viral illnesses that do not benefit from antibiotic treatment and promoting alternative symptom management strategies, such leaflets can help prevent unnecessary use of antibiotics for conditions such as uncomplicated acute respiratory tract infections (ARTIs) like colds and viral sore throats. In turn, this reduces pressure on healthcare providers to prescribe antibiotics unnecessarily. Studies have shown that antibiotic-free leaflets can significantly reduce patient expectations for antibiotics, supporting a more responsible approach to prescribing [15].

The understanding that antibiotics are not always necessary and also may have adverse effects (and cost money), and that a high societal use promotes a rapid increase in AMR, is a premise for the patient to not automatically wish for antibiotics. Additionally, it creates the

context to interpret the CRP POCT result: normal and mildly elevated values means that antibiotics are generally unnecessary and may be safely withheld.

We will address these challenges in the 2CARE study:

1) an adequate understanding of the AMR context may work together with the use of CRP POCT to additionally lower antibiotic use. Hence, this understanding needs to be instilled in the patient. To aid in this, the use of written materials (PILs) in the consultation is evaluated in the present study.

2) What CRP-threshold to choose: a low threshold may not lower antibiotic use much, while a high threshold could significantly reduce antibiotic use, but possibly at the expense of a higher rate of adverse events (hospitalisations, longer recovery times) due to the risk of undertreatment of bacterial pneumonia. In order to identify relevant thresholds, guidelines are reviewed to assess which CRP values are applied in adult patients who present with acute respiratory infection.

METHODS

Aim

The study aims to determine how CRP levels and PILs affects antibiotic prescribing in adults presenting with acute respiratory symptoms in Kyrgyz primary care.

Objectives

1. To evaluate the effectiveness of PILs to reduce the participants' tendency to take antibiotics and the safety of PILs with respect to not increase hospitalisations.
2. To evaluate the effectiveness and safety of selected CRP thresholds (low, middle, high) for adults with acute respiratory tract infection.

Study design

A multicentre, open-label, individually randomised controlled clinical trial with 21 days blinded follow-up comparing PIL to no PIL in a 1:1 ratio on antibiotic use and safety. Additionally, orthogonally, we will assess antibiotic use and safety across three CRP cut-offs (20-40-60mg/L) in a 1:1:1 ratio to determine the optimal CRP cut-offs in these cohorts. Thus, in total six groups will be randomly formed and compared. The trial will be conducted in 14 selected district primary-level healthcare centres (HCs) in rural Kyrgyzstan and one urban primary-level HC in Bishkek – 15 centres in total.

Study setting

Some of the centres will be drawn from the COORDINATE study, while others will be newly established. New HCs will be selected pragmatically, considering the logistics of the study, the distance of the study sites from each other and the transport infrastructure for the convenience of the researcher to get there. One HC in Bishkek will be chosen pragmatically, taking into account its convenient location, large patient flow and the administration’s loyal attitude towards the research group.

The HCWs will primarily be from the primary healthcare level, including family doctors or family nurses. In many facilities, there is no doctor available, and patients are treated solely by a feldsher. These HCs are small rural primary health clinics with only the most basic equipment; for instance, very few of them have the necessary tools for conducting basic blood counts or urinalysis.

HCWs from newly included HCs will be trained in conducting CRP tests and interpreting the results, while HCWs from the HCs participating in the COORDINATE study will be given opportunity to refresh their skills and knowledge. Training of new study sites’ HCWs, as in the COORDINATE RCT, will be conducted by the research team at the fields in the State (Kyrgyz) or Official (Russian) languages at the discretion of the HCWs themselves. Healthcare workers (HCWs) will be trained to use the Aidian QuickRead Go device and interpret its results. Unlike the COORDINATE training, this programme will include training HCWs to provide PIL only to participants in Group A (AX, AY and AZ), which will be communicated to the HCW by the study team in advance. Additionally, the training will cover the threshold values of CRP.

In this study, we will combine old study sites with newly selected ones. By integrating both old and new study sites, we can better understand the impact of varied factors on healthcare delivery and outcomes. These differences will be addressed in the covariate section, where we will define and analyse the influence of site-specific characteristics. Additionally, a subgroup analysis will be conducted to further explore the distinctions between the old and new sites, ensuring a thorough and nuanced understanding of the study results.

Participants

Adult patients aged 18 to 70 years with respiratory symptoms who visit any of the 15 selected primary HCs for two winter and early spring periods (2025 and 2026) during normal working hours will be screened for eligibility on weekdays by a research assistant (RA) prior to routine consultation by a HCW.

Eligibility criteria

All eligible participants must provide a written informed consent before being enrolled in the study.

Inclusion criteria

Patients meeting the following criteria will be included in the study:

- Adults aged 18 to 70 years with ARTI according to the attending HCW;
- Having at least one of the following respiratory symptoms lasting for less than 2 weeks (with or without a fever):
  - cough;
  - shortness of breath;
  - sore throat;
  - stuffy nose;
  - wheezing.
- Able and willing to comply with all study requirements.
- Able and willing to give informed consent.

Exclusion criteria

- Severely ill and in need of urgent referral where measurement of POCT CRP is not relevant or would delay the process;
- Terminally ill patients;
- Patients with known immunosuppression or severe chronic disease (HIV, liver failure, kidney failure, history of neoplastic disease, long term systemic steroid use or similar conditions as assessed by the health worker or research team);
- Patients who are not able to participate in follow-up procedures (lack of telephone etc.);
- Have taken antibiotics within 24 hours before the index consultation
- Pregnant women
- Unable or unwilling to provide informed consent.

Study procedures

The research team will screen all patients presenting to the healthcare centres to identify eligible participants. Eligible participants will be asked by the research team to sign an informed consent form after information about the study and its procedures. The informed consent is available in written form as well as oral information provided by research team. Patients of both sexes and any ethnicity will be included in the study. All included patients will complete the case report form (CRF). The age limit of up to 70 years was chosen in connection with the average life expectancy in Kyrgyzstan in 2022 - 72 years [16]. Patients above this age may be particular frail.

When a patient is enrolled in the study, the research team in each study site will contact by telephone an investigator at the central office (National Centre of Cardiology and Internal Medicine named after academician M. Mirrakhimov, Bishkek, Kyrgyz Republic), who will not know the name or clinical status of the participant, to provide the unique participant identification number, randomise and allocate the participant to one of the six groups.

All further actions will take place in the HCW's office. The medical worker himself will be in the office (if the main medical worker is a physician or feldsher, he/she often has a nurse as an assistant).

Intervention

Consenting participants will be randomised to one of six intervention groups:

- group AX (CRP + PILs; CRP cut-off 20 mg/l),
- group AY (CRP + PILs; CRP cut-off 40 mg/l),
- group AZ (CRP + PILs; CRP cut-off 60 mg/l),
- group BX (CRP with cut-off 20 mg/l),
- group BY (CRP with cut-off 40 mg/l) and
- group BZ (CRP with cut-off 60 mg/l).

To achieve this, stratified block randomisation lists will be created for each study site.

Participants in all six groups will undergo the CRP POCT test during a consultation with a HCW who will be pre-trained in the operation of the machine and interpretation of the results. The kit used will be the Aidian (Espoo, Finland) QuickRead go CRP POCT [17]. According to the cut-off subgroup to which the participant is assigned, the decision to prescribe an antibiotic will be made according to the cut-off (>20 mg/l, >40 mg/l and >60 mg/l). The result will be recorded in the CRF by the study team and explained to the patient in plain language by the HCW (Kyrgyz or Russian).

Unlike Groups B (BX, BY and BZ), where only the CRP test will be performed, in groups A (AX, AY and AZ), in addition to the CRP, the HCW will also hand the participant the PIL, regardless of whether an antibiotic is prescribed or not. If the participant has been prescribed other medications, including pyrolytics or immunomodulators, the PIL will also be given. When giving the PIL, the HCW explains to the participant the importance of not prescribing antibiotics to patients without obvious signs of bacterial infection, when CRP levels are low and viral infection is suspected.

Following the baseline consultation, follow-up calls will be made to the telephone number provided by the participants on days 3, 7, 14 and 21 (Figure 1) by a blinded researcher from the Bishkek office.

Beyond the information written in the PIL, which half of the participants will receive, all will be informed that if symptoms worsen or fail to improve, they need to contact health services again; this is usual management of patients with ARTI symptoms in Kyrgyz primary care.

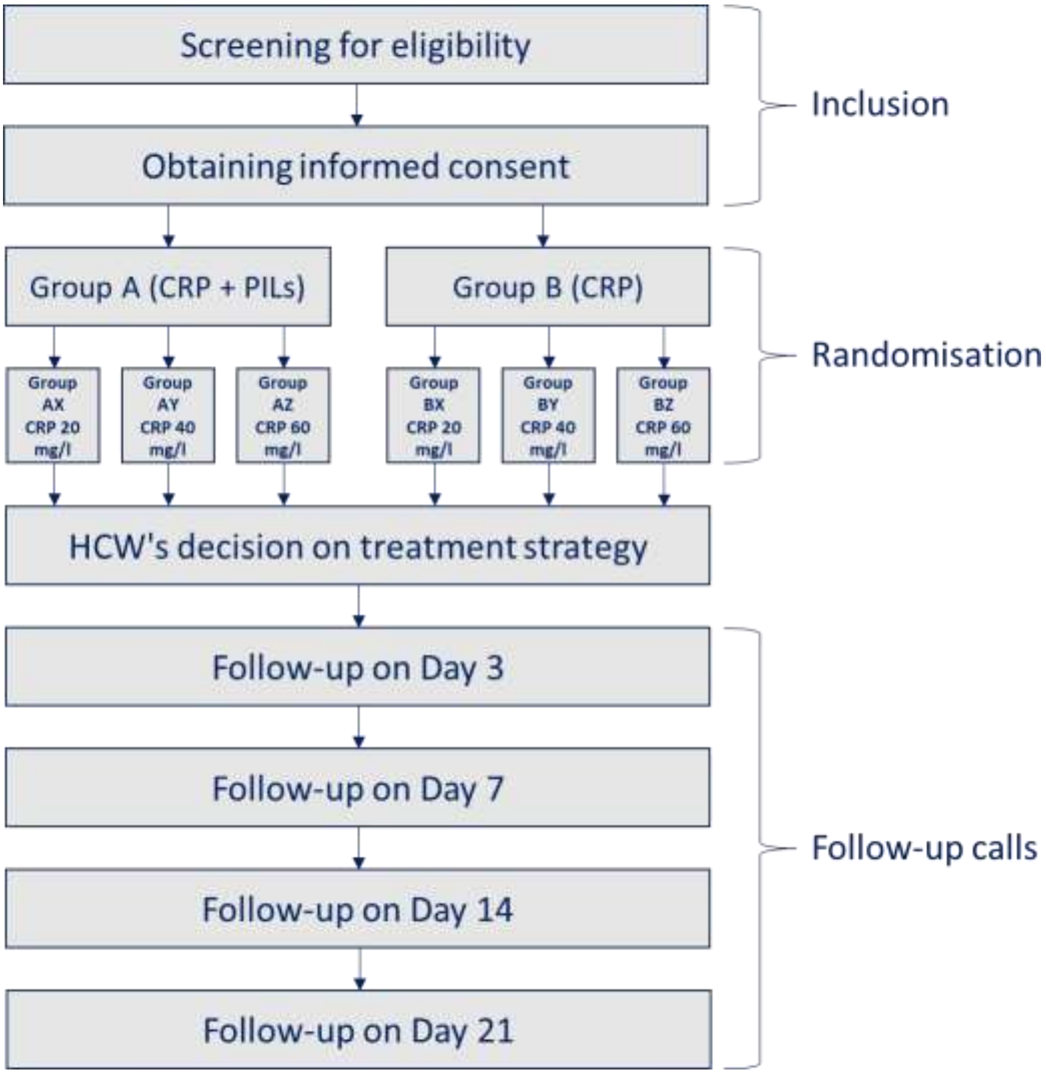

Figure 2. The study flowchart.

Criteria for discontinuing or modifying allocated interventions

Each participant is expected to participate in the trial for 21 days (Figure 2) to report any adverse events (AE). The trial will stop on the 21st day of the last enrolled participant. Serious (S)AEs will be reported by the HCWs or RAs and reviewed by the Safety Board consisting of Primary investigator (non-voter), Biostatistician, and three academic members (one Kyrgyz and two Danish). SAE notification is done to the Primary investigator by via e-mail or phone within 24 hours. All SAEs must be reported to the Safety Board within 14 days. The Safety board retains the right to end the trial at any time due to *safety concerns.* If the trial is ended prematurely, the PI and Kyrgyz team are responsible for informing all participants and planning adequate follow-up.

Discontinuation from the trial could occur due to the following reasons:

- Withdrawn consent.

- Other violations of the protocol.

The participants will be informed that they are not obliged to provide a reason for discontinuation. If an AE is a contributory factor to the discontinuation, it will be followed up appropriately. Regardless of any discontinuation, the participants should, if possible, be retained in the study for follow-up. Data until discontinuation will be included in the analyses. Leaving participants will not be replaced by new participants. If an exclusion criterion is met during the 21-day follow-up period, it will not automatically lead to discontinuation.

Outcomes

The primary outcome (mainly relevant relative to the PIL intervention) is total antibiotic use (prescribed as well as non-prescribed (e.g. purchased over the counter or taken from home storage) over 21 days.

The second primary outcome (mainly relevant relative to the CRP threshold intervention) is safety measured as hospital admission over 21 days.

Secondary study outcomes are: antibiotics prescribed at the baseline assessment; self-reported recovery; mortality; antibiotic use started outside of health care visits; antiviral treatment; re-consultation; hospital referral at the baseline consultation.

Sample size and power calculation

We expect 40% of participants to use antibiotics (AB) within 21 days without the PIL. A 10 percentage point reduction to 30% with PIL is considered a relevant effect. For binary outcomes, detecting this difference with 90% power and 5% significance in a t-test for binary outcomes requires 506 participants per arm. Total sample size for the superiority outcome on antibiotic reduction is 1012 participants.

The safety outcome (hospitalisation) is expected to be similar across the CRP threshold groups at 5%, and a difference of 5 percentage points (the non-inferiority margin) is the largest acceptable difference; and would make us seriously question the safety of this approach as it will matter in practice. Then, to conclude that one of the groups is not inferior to another group, i.e. the difference reliably does not exceed 5 percentage points, with 90% power and 5% significance in a t-test (for binary outcomes), we need 326 participants in each arm. Total sample size for the non-inferiority outcome 978 participants.

To cover both primary outcomes, we need ~1000 participants. Based on the COORDINATE trial a high follow-up rate may be expected, but we will allow up to 5% loss to follow-up in this adult population. Therefore, the planned sample will be 1050 participants.

Statistical methods

The study population and randomisations are investigated in two Tables 1 (which are combined into one) where the distributions of a set of baseline characteristics are compared between the randomisation groups; separately for the PIL intervention and for the CRP intervention. The baseline characteristics include: living place, age, sex, occupation etc. Continuously valued characteristics are shown as medians with inter-quartile range (IQR), and tested with a Kruskal-Wallis non-parametric test, categorically valued variables are shown as number with percentage for each category, and tested with chi-squared test.

The outcomes are all devised as indicator variables of whether a certain event has occurred up to the follow-up time point. The difference in outcome incidence – a risk difference (RD) – between randomisation groups at each follow-up time point is estimated in linear regression models with binomial error term. The inherent excess correlation between outcomes on the same person at different follow-up time points is adjusted for with the method of Generalised Estimating Equations (GEE). Parallel we will assess the quotient in outcome incidence – an odds ratio (OR) – between randomisation groups at each follow-up time point in a logistic regression model. Also, here the excess correlation is adjusted for through GEE. The effects of the two randomisations are assessed separately, but in the assessment of one, we adjust for the other randomisation and vice versa.

Interim analysis: To determine if any intervention is clearly beneficial or harmful compared to the concurrent control, or the trial is otherwise statistically obviously futile the trial may be stopped early. The interim analysis is a pre-defined analysis of an incomplete data set while the study is on-going, which we plan to conduct after 500 inclusions (50% of target inclusion).

We will investigate some subgroup analyses: 1) age group: 18-44/45-59/60-70, 2) urban/rural, 3) Type of Health clinic, 4) male/female.

DISCUSSION

RCTs are rare in Kyrgyzstan. However, our team has successfully completed a RCT in children with ARTI using a similar CRP guidance approach (COORDINATE). As such we know this approach is feasible, omitting the need for a pilot trial. We will replicate much of the organisational and training developed in the COORDINATE trial. The new trial will include adults with chronic diseases and we choose to focus more on the safety aspect in order to avoid missing potential serious adverse effects. This includes hospitalisations that are relatively common in Kyrgyzstan, occurring at an estimated 5% level in controls. We choose to accept a five percentage point increase in hospitalisations. HCW not issuing antibiotic prescriptions in accordance with CRP guidance may however be more prone to admit a patient to hospital that does not rapidly recover or worsen. This to not overlook a potential serious complication.

TRIAL STATUS

Protocol version 2.1. December 2nd, 2024. Date of start recruitment estimated to be January 2025. Estimated end of recruitment May 2025.

DECLARATIONS

Ethics approval and consent to participate

Ethical approval for the 2CARE RCT was obtained during a meeting of the Ethics Committee, which included the chairman and other members of the Ethics Committee of the National Centre of Cardiology and Internal Medicine named after academician M. Mirrakhimov. During the meeting, the study protocol was reviewed, and the principal investigator addressed questions regarding the intervention. The decision was documented and signed by each member (Ref. No 10 27.11.2024). As stated earlier, before inclusion in the study, the participant will be asked to sign an informed consent, otherwise the participant cannot be included in the study. The informed consent is on the first page of the CRF.

Competing interests

There are no competing interests.

Funding

Please see attached budget. The cost of the project described above is budgeted to 197.800 USD. There is currently room in the already approved budget of 70.000 USD due to changes in methodology for the microbiological analyses relating to the COORDINATE trial. Therefore, the project applies for an extension of funding of 127.800 USD for the project in Kyrgyzstan and 21.520 USD for SMEs.

REFERENCES

1. O'Neill J. Tackling drug-resistant infections globally: Final report and recommendations. Review on Antimicrobial Resistance. 2016. Available from: https://pubmed.ncbi.nlm.nih.gov
2. GBD 2019 Antimicrobial Resistance Collaborators. Global burden of bacterial antimicrobial resistance in 2019: a systematic analysis. Lancet. 2022;399(10325):629-55. doi:10.1016/S0140-6736(21)02724-0.
3. Holmes AH, Moore LS, Sundsfjord A, Steinbakk M, Regmi S, Karkey A, et al. Understanding the mechanisms and drivers of antimicrobial resistance. Lancet. 2016;387(10014):176-87. doi:10.1016/S0140-6736(15)00473-0.
4. Beyene T, Abdissa A, Bedada T, Abera A. Multidrug-resistant bacterial isolates and associated factors in the teaching hospital, Central Ethiopia. PLoS One. 2021;16(4):e0251283. doi:10.1371/journal.pone.0251283.
5. Baktygul, K., Marat, B., Ashirali, Z., Harun-Or-Rashid, M. & Sakamoto, J. An Assessment of antibiotics prescribed at the secondary health-care Level in the Kyrgyz Republic. Nagoya J. Med. Sci. 73, 157 (2011).
6. World Health Organization Regional Office for Europe. Central Asian and European Surveillance of Antimicrobial Resistance: annual report 2020. Copenhagen: World Health Organization; 2020. (WHO/EURO:2020-3469-43228-60585). Available from: https://www.who.int/europe/publications/i/item/WHO-EURO-2020-3469-43228-60585
7. World Health Organization. Antimicrobial resistance: global report on surveillance. Geneva: World Health Organization; 2014. 257 p. ISBN: 9789241564748. Available from: https://www.who.int/publications/i/item/9789241564748
8. Nurmatov Z. Influenza and ARVI in Kyrgyzstan. Russ J Infect Immun. 2015;4:365–374. doi:10.15789/2220-7619-2014-4-365-374
9. Isaeva E, Bloch J, Akylbekov A, Skov RL, Poulsen A, Kurtzhals JAL, Reventlow S, Sreenivasan N, Mademilov M, Siersma VD, Sooronbaev T, Kjærgaard J, Aabenhus RM. C-reactive protein testing in primary care and antibiotic use in children with acute respiratory tract infections in Kyrgyzstan: an open-label, individually randomised, controlled trial. Lancet Reg Health Eur. 2025 Jan 10;51:101184. doi: 10.1016/j.lanepe.2024.101184. PMID: 39886015; PMCID: PMC11774811.
10. Do NTT, Vu TVD, Greer RC, Dittrich S, Vandendorpe M, Pham NT, Ta DN, Cao HT, Khuong TV, Le TBT, Duong TH, Nguyen TH, Cai NTH, Nguyen TQT, Trinh ST, van Doorn HR, Lubell Y, Lewycka S. Implementation of point-of-care testing of C-reactive protein concentrations to improve antibiotic targeting in respiratory illness in Vietnamese primary care: a pragmatic cluster-randomised controlled trial. Lancet Infect Dis. 2023 Sep;23(9):1085-1094. doi: 10.1016/S1473-3099(23)00125-1. Epub 2023 May 22. PMID: 37230105.
11. Smedemark SA, Aabenhus R, Llor C, Fournaise A, Olsen O, Jørgensen KJ. Biomarkers as point-of-care tests to guide prescription of antibiotics in people with acute respiratory infections in primary care. Cochrane Database Syst Rev. 2022 Oct

17;10(10):CD010130. doi: 10.1002/14651858.CD010130.pub3. PMID: 36250577; PMCID: PMC9575154.
12. Ngwengi, Yembu et al. “Evaluation of CRP as a marker for bacterial infection and malaria in febrile children at the Douala Gyneco-Obstetric and Pediatric Hospital.” PloS one vol. 18,7 e0289012. 21 Jul. 2023, doi:10.1371/journal.pone.0289012
13. Escadafal C, Incardona S, Fernandez-Carballo BL, et alThe good and the bad: using C reactive protein to distinguish bacterial from non-bacterial infection among febrile patients in low-resource settingsBMJ Global Health 2020;5:e002396.
14. Sustersic M, Gauchet A, Foote A, Bosson JL. How best to use and evaluate Patient Information Leaflets given during a consultation: a systematic review of literature reviews. Health Expect. 2017 Aug;20(4):531-542. doi: 10.1111/hex.12487. Epub 2016 Sep 26. PMID: 27669682; PMCID: PMC5512995.
15. de Bont EGPM, Alink M, Falkenberg FCJ, et al. Patient information leaflets to reduce antibiotic use and reconsultation rates in general practice: a systematic review. BMJ Open 2015;5:e007612. doi: 10.1136/bmjopen-2015-007612
16. World Health Organization. WHO country profile: Kyrgyzstan. Geneva: World Health Organization; 2024. Available from: https://data.who.int/countries/417
17. Aidian. QuikRead Go: general information. 2024. Available from: https://www.aidian.eu/point-of-care/quikread-go/quikread-go-instrument
